# Nonpasteurized Dairy Products, Disease Outbreaks, and State Laws—United States, 1993–2006

**DOI:** 10.3201/eid1803.111370

**Published:** 2012-03

**Authors:** Adam J. Langer, Tracy Ayers, Julian Grass, Michael Lynch, Frederick J. Angulo, Barbara E. Mahon

**Affiliations:** Centers for Disease Control and Prevention, Atlanta, Georgia, USA

**Keywords:** dairy products, nonpasteurized, unpasteurized, foodborne illnesses, disease outbreaks, regulations, Campylobacter, Salmonella, Escherichia coli, Shigella, Listeria, Brucella, bacteria

## Abstract

Most dairy-associated outbreaks occurred in states that permitted sale of these products.

## Medscape CME Activity

Medscape, LLC is pleased to provide online continuing medical education (CME) for this journal article, allowing clinicians the opportunity to earn CME credit.

This activity has been planned and implemented in accordance with the Essential Areas and policies of the Accreditation Council for Continuing Medical Education through the joint sponsorship of Medscape, LLC and Emerging Infectious Diseases. Medscape, LLC is accredited by the ACCME to provide continuing medical education for physicians.

Medscape, LLC designates this Journal-based CME activity for a maximum of 1 *AMA PRA Category 1 Credit(s)*^TM^. Physicians should claim only the credit commensurate with the extent of their participation in the activity.

All other clinicians completing this activity will be issued a certificate of participation. To participate in this journal CME activity: (1) review the learning objectives and author disclosures; (2) study the education content; (3) take the post-test with a 70% minimum passing score and complete the evaluation at www.medscape.org/journal/eid; (4) view/print certificate.

**Release date: February 21, 2012; Expiration date: February 21, 2013**

## Learning Objectives

Upon completion of this activity, participants will be able to:

- Evaluate the epidemiology of foodborne illness related to the consumption of dairy products
- Analyze the clinical presentation and outcomes of foodborne disease related to the consumption of dairy products
- Distinguish the organism most commonly associated with foodborne illness after consumption of unpasteurized dairy products
- Assess sources of contamination of pasteurized dairy products

## CME Editor

**P. Lynne Stockton, VMD, MS, ELS(D),** Technical Writer/Editor, *Emerging Infectious Diseases. Disclosure: P. Lynne Stockton, VMD, MS, ELS(D), has disclosed no relevant financial relationships.*

## CME Author

**Charles P. Vega, MD,** Health Sciences Clinical Professor; Residency Director, Department of Family Medicine, University of California, Irvine. *Disclosure: Charles P. Vega, MD, has disclosed no relevant financial relationships.*

## Authors

*Disclosures:* ***Adam J. Langer, DVM, MPH; Tracy Ayers, MS; Julian Grass, MPH; Michael Lynch, MD, MPH; Frederick J. Angulo, DVM, PhD;**** and ****Barbara E. Mahon, MD, MPH, ****have disclosed no relevant financial relationships.*
