# Supplementary material for: Nonpasteurized Dairy Products, Disease Outbreaks, and State Laws—United States, 1993–2006
Source: Emerg Infect Dis. 2012 Mar;18(3):385–91. doi: 10.3201/eid1803.111370 (PMC3309640; doi:10.3201/eid1803.111370)
Supplement: Related texts

# Research article

**DOI:** 10.3201/eid1803.111370

## Abstract

Although pasteurization eliminates pathogens and consumption of nonpasteurized dairy products is uncommon, dairy-associated disease outbreaks continue to occur. To determine the association of outbreaks caused by nonpasteurized dairy products with state laws regarding sale of these products, we reviewed dairy-associated outbreaks during 1993–2006. We found 121 outbreaks for which the product’s pasteurization status was known; among these, 73 (60%) involved nonpasteurized products and resulted in 1,571 cases, 202 hospitalizations, and 2 deaths. A total of 55 (75%) outbreaks occurred in 21 states that permitted sale of nonpasteurized products; incidence of nonpasteurized product–associated outbreaks was higher in these states. Nonpasteurized products caused a disproportionate number (≈150× greater/unit of product consumed) of outbreaks and outbreak-associated illnesses and also disproportionately affected persons <20 years of age. States that restricted sale of nonpasteurized products had fewer outbreaks and illnesses; stronger restrictions and enforcement should be considered.

In the United States, milk and other dairy products are dietary staples; the 2010 Dietary Guidelines for Americans recommend that most Americans include dairy products in their diet (*1*). However, numerous pathogens can contaminate dairy products and cause illness and death. Milkborne infections were relatively common before the advent of pasteurization in the late 19th century (*2*), and in the United States today, illness related to consumption of nonpasteurized dairy products remains a public health problem.

In 1948, Michigan enacted the first statewide requirement that dairy products be pasteurized, and many other states soon did the same (*2*). In 1987, the United States Food and Drug Administration prohibited distribution of nonpasteurized dairy products in interstate commerce for sale to consumers (*3*). However, sale of nonpasteurized dairy products within the state where they are produced is regulated by each state, and some states permit sale of these products. Despite the federal ban on the sale of nonpasteurized products in interstate commerce, the broad use of pasteurization by the dairy industry, and the infrequency with which nonpasteurized dairy products are consumed, illnesses and outbreaks associated with consumption of these products continue to occur (*4**–**23*).

State and local health departments report foodborne disease outbreaks to the Centers for Disease Control and Prevention (CDC) through the Foodborne Disease Outbreak Surveillance System. As a result of efforts to enhance outbreak surveillance starting in 1998, the total number of outbreak reports increased substantially (*24*). A recent comprehensive analysis of foodborne disease outbreaks associated with dairy products (dairy-associated outbreaks) reported to CDC reviewed outbreaks that occurred during 1973–1992 (*4*). We reviewed subsequent dairy-associated outbreaks, reported in the United States during 1993–2006. We characterized the outbreaks and examined their association with state laws regarding sale of nonpasteurized dairy products.

## Methods

To compare the incidence of foodborne outbreaks involving nonpasteurized dairy products among states with differing laws with regard to the sale of these products (i.e., states that permitted their sale vs. states that prohibited their sale), we reviewed reports of foodborne disease outbreaks involving dairy products reported to CDC during 1993–2006. These reports, completed by state and local health departments, typically included the number of cases associated with the outbreak; the age and sex distribution of outbreak-associated case-patients; the number of hospitalizations and deaths; the etiologic agent associated with the outbreak; the type of dairy product implicated (e.g., fluid milk, cheese); and whether the implicated dairy product was marketed, labeled, or otherwise presented to the consumer as pasteurized or nonpasteurized. Hereafter, we refer to these products as pasteurized or nonpasteurized. Thus, any outbreak involving a dairy product that was contaminated after pasteurization or that was intended to be pasteurized but underwent inadequate pasteurization was classified as involving pasteurized product. When possible, we corrected missing or incomplete data by asking the health department that conducted the investigation for more information.

To determine whether the sale of nonpasteurized dairy products was legal at the time of each outbreak, we contacted the 50 state departments of health and agriculture and requested data on whether the state permitted the sale of nonpasteurized dairy products produced in that state for each year from 1993 through 2006. We defined an illegal state-year as a year in which a state prohibited the sale of all nonpasteurized products, and we defined a legal state-year as a year in which a state permitted the sale of nonpasteurized dairy products produced in that state. Data on the estimated population, by state, for each year were obtained from the US Census Bureau. To compare the incidence of outbreak and outbreak-associated cases during illegal state-years to that during legal state-years, we stratified the outbreaks by legal status of the state in which the outbreak occurred at the time of the outbreak and calculated incidence density ratios for reported outbreaks (Poisson model) and for outbreak-associated cases (zero-inflated negative binomial model).

## Results

During 1993–2006, a total of 30 states reported 122 foodborne disease outbreaks caused by contaminated dairy products. Dairy-associated outbreaks occurred in all years except 1996, and outbreaks involving nonpasteurized dairy products occurred in all years except 1994 and 1996. The number of reported dairy-associated outbreaks increased in 1998 after surveillance for foodborne disease outbreaks was enhanced (Figure 1).

**Figure 1:** Number of dairy product–associated outbreaks, by year and pasteurization status of product, United States, 1993–2006.

Whether the product was pasteurized or nonpasteurized was known for 121 of the 122 outbreaks, and most outbreaks (73 [60%]) involved nonpasteurized dairy products. Of the 121 outbreaks for which product pasteurization status was known, 65 (54%) involved cheese and 56 (46%) involved fluid milk. Of the 65 outbreaks involving cheese, 27 (42%) involved cheese made from nonpasteurized milk. Of the 56 outbreaks involving fluid milk, an even higher percentage (82%) involved nonpasteurized milk.

The 121 outbreaks involving dairy products for which pasteurization status was known resulted in 4,413 reported illnesses. Among these illnesses, 1,571 (36%) resulted from nonpasteurized dairy products. The median number of persons reported ill during outbreaks involving nonpasteurized dairy products was 11 (range 2–202). Outbreaks involving nonpasteurized dairy products resulted in 202 hospitalizations (hospitalization rate 13%). In contrast, outbreaks involving pasteurized dairy products resulted in 37 hospitalizations (hospitalization rate 1%). Two deaths were associated with an outbreak caused by consuming nonpasteurized dairy products, and 1 death was associated with an outbreak caused by a pasteurized product (Table).

**Table:** Characteristics of disease outbreaks after consumption of dairy products, United States, 1993–2006

Product	Outbreak characteristic, no.			
	Total	Associated illnesses	Associated hospitalizations	Associated deaths
Nonpasteurized				
Fluid milk	46	930	71	0
Cheese	27	641	131	2
Total	73	1,571	202	2
Pasteurized				
Fluid milk	10	2,098	20	0
Cheese	38	744	17	1
Total	48	2,842	37	1
All dairy	121	4,413	239	3

Ill persons in outbreaks involving nonpasteurized dairy products were generally younger than those in outbreaks involving pasteurized dairy products. For the 60 outbreaks involving nonpasteurized dairy products for which age of patients was known, 60% of patients were <20 years of age; for the 37 outbreaks involving pasteurized dairy products for which age of patients was known, 23% of patients were <20 years of age (p<0.001).

The causative agent was identified for all 73 outbreaks involving nonpasteurized dairy products; all were caused by bacteria. One outbreak was caused by *Campylobacter* spp. and Shiga toxin–producing *Escherichia coli*. Among the remaining 72 outbreaks, 39 (54%) were caused by *Campylobacter* spp., 16 (22%) by *Salmonella* spp., 9 (13%) by Shiga toxin–producing *E. coli*, 3 (4%) by *Brucella* spp., 3 (4%) by *Listeria* spp., and 2 (3%) by *Shigella* spp. Among the 30 outbreaks involving pasteurized dairy products for which the causative agent was reported, 13 (44%) were caused by norovirus, 6 (20%) by *Salmonella* spp., 4 (13%) by *Campylobacter* spp., 3 (10%) by *Staphylococcus aureus*, and 1 (3%) each by *Clostridium perfringens*, *Bacillus cereus*, *Listeria* spp., and *Shigella* spp.

A total of 48 reported outbreaks involved pasteurized dairy products. The source of contamination was reported for 7 (14%) of these outbreaks, of which at least 4 (57%) probably resulted from post-pasteurization contamination by an infected food handler. Failure of the consumer to store the dairy product at an appropriate temperature probably contributed to 3 other outbreaks. Such temperature abuse can enable pathogens (present because they either survived pasteurization in low numbers or were introduced after pasteurization) to multiply to concentrations capable of causing illness.

During the study period, 43 (86%) states did not change their legal status regarding the sale of nonpasteurized dairy products produced in that state. Among these 43 states, selling nonpasteurized dairy products produced in that state was legal in 21 (49%). Of the 7 states that changed their legal status, 3 changed from legal to illegal (Mississippi in 2005, Ohio in 2003, and Wisconsin in 2005), 3 changed from illegal to legal (Arkansas in 2005, Illinois in 2005, and Nevada in 2005), and 1 (Oregon) changed from legal to illegal in 1999 and then back to legal in 2005 (Figure 2).

**Figure 2:** Legal status of nonpasteurized dairy product sale or distribution, by state, United States, for A) 1993, B) 1999, and C) 2006. Gray shading indicates states where nonpasteurized dairy product sale or distribution was permitted. States outlined in black changed legal status during the study period.

Among the 700 state-years (14 years × 50 states) included in our analysis of the association of legal sales status and nonpasteurized dairy–associated outbreaks, sale of nonpasteurized dairy products produced in the state was legal for 342 state-years and illegal for 358 state-years. We excluded from analysis 2 outbreaks caused by nonpasteurized dairy products because each occurred in multiple states with differing laws. Of the 71 remaining outbreaks involving nonpasteurized dairy products, 55 (77%) occurred in states where sale of nonpasteurized dairy products produced in that state was legal. Among these 71 outbreaks involving nonpasteurized dairy products, 1,526 persons became ill and 1,112 (73%) of these illnesses occurred in states where it was legal to sell nonpasteurized dairy products. Also among these 71 outbreaks involving nonpasteurized dairy products, 15 occurred in states where sale of nonpasteurized dairy products was illegal. The source of the nonpasteurized dairy products was reported for 9 of these outbreaks: 7 (78%) were associated with nonpasteurized dairy products obtained directly from the producing dairy farm, 1 was associated with nonpasteurized dairy products obtained under a communal program to purchase shares in dairy cows (i.e., cow shares, a scheme used to circumvent state restrictions on commercial sales of nonpasteurized dairy products) (*11*), and 1 was limited to members of a large extended family who consumed nonpasteurized milk from their own cow.

Incidence density ratios (IDRs) for nonpasteurized product–associated outbreaks and outbreak-associated cases during legal and illegal state-years varied by the type of dairy product (milk or cheese) and are reported separately. In states where it was legal to sell nonpasteurized dairy products, the rate of outbreaks caused by nonpasteurized fluid milk was >2× as high as in states where it was illegal to sell nonpasteurized dairy products (IDR 2.20, 95% CI 1.14–4.25). The rate of outbreak-associated illnesses caused by nonpasteurized fluid milk was 15% higher in states where it was legal to sell nonpasteurized dairy products, but this result was not statistically significant (IDR 1.15, 95% CI 0.24–5.54). States where it was legal to sell nonpasteurized dairy products had nearly 6× the rate of outbreaks caused by cheese made from nonpasteurized milk (IDR 5.70, 95% CI 1.71–19.05) and nearly 6× the rate of outbreak-associated illnesses (IDR 5.77, 95% CI 0.59–56.31), although the IDR for outbreak-associated illnesses was not statistically significant.

## Discussion

Incidence of outbreaks caused by nonpasteurized dairy products was higher in states that permitted the sale of nonpasteurized dairy products than in states that prohibited such sale. This association was evident for nonpasteurized fluid milk and cheese made from nonpasteurized milk. Although this association did not extend to the rates of outbreak-associated cases, factors other than whether it was legal to sell nonpasteurized dairy products probably affect the number of cases that occur in an outbreak. These factors include the volume and area of distribution of the contaminated product, the pathogen involved, the underlying health status of the exposed persons, and the ability of the responding public health agency to swiftly intervene to terminate the outbreak.

Because consumption of nonpasteurized dairy products is uncommon in the United States, the high incidence of outbreaks and outbreak-associated illness involving nonpasteurized dairy products is remarkable and greatly disproportionate to the incidence involving dairy products that were marketed, labeled, or otherwise presented as pasteurized. In a population-based survey conducted in 1996–1997, only 1.5% of respondents reported having consumed nonpasteurized dairy products in the 7 days before being interviewed; and in the 2003–2004 and 2005–2006 National Health and Nutrition Examination Surveys, only <1% of respondents who drank milk reported that they usually drank nonpasteurized milk (*21**,**25**,**26*). Because many of these respondents also reported consuming pasteurized dairy products, the proportion of dairy products consumed nonpasteurized by volume or weight is probably <1%. To illustrate this point, it is useful if we provide a hypothetical weighting of the findings in this study by the amount of nonpasteurized and pasteurized dairy products consumed. Total milk production in the United States in 2010 was estimated at 193 billion pounds, suggesting that ≈2.7 trillion pounds of milk were consumed during the 14 years from 1993 through 2006 (*27*). If 1% of dairy products were consumed nonpasteurized, then during these 14 years, 73 outbreaks were caused by the 27 billion pounds of nonpasteurized dairy products that were consumed and 48 by the 2,673 billion pounds of pasteurized products that were consumed. Therefore, the incidence of reported outbreaks involving nonpasteurized dairy products was ≈150× greater, per unit of dairy product consumed, than the incidence involving pasteurized products. If, as is probably more likely, <1% of dairy products are consumed nonpasteurized, then the relative risk per unit of nonpasteurized dairy product consumed would be even higher.

After 1998, when surveillance for foodborne outbreaks was enhanced, the number of reported foodborne disease outbreaks caused by dairy products increased, as did the total number of reported foodborne outbreaks. Outbreaks involving nonpasteurized dairy products were all associated with bacterial enteric pathogens, most of which have known animal reservoirs. In contrast, among outbreaks in which a pasteurized dairy product was implicated, the most commonly reported causative agent was norovirus (44% of outbreaks), a pathogen with a human reservoir. These results suggest that outbreaks caused by nonpasteurized dairy products are probably caused by pathogens in the dairy environment, which would be eliminated by proper pasteurization, and that outbreaks caused by pasteurized dairy products are probably caused by contamination of the products at some point after pasteurization.

The objective of pasteurization is to eliminate from fluid milk those pathogens that originate in the dairy environment; however, pasteurization does not protect against contamination that might occur later, such as during food handling. In addition, if pasteurization is not performed properly (for appropriate times and at appropriate temperatures), pathogens might not be eliminated from the milk. Appropriate post-pasteurization food-handling practices can minimize the risk for reintroduction of pathogens into dairy products after pasteurization. In addition, other precautions, such as maintaining the dairy product at an appropriate temperature and disposing of expired products, reduce the risk to the consumer should the product become contaminated after pasteurization. When outbreaks do occur because of contamination of dairy products that are marketed as pasteurized, the source of contamination is typically traced to improper pasteurization, improper storage, or improper handling of the products after marketing (*28**–**30*). In our study, all outbreaks associated with pasteurized products for which information on the source of contamination was available were attributed to post-pasteurization mishandling.

Among outbreak-associated cases involving nonpasteurized dairy products, 60% involved persons <20 years of age. Public health and regulatory authorities are obligated to protect persons who cannot make fully informed decisions (e.g., children) from potential health hazards. Dietary decisions for younger children, in particular, are often made by caregivers. The American Academy of Pediatrics advises against giving nonpasteurized dairy products to children and recommends that pediatricians counsel caregivers against use of these products (*31*).

Proportionately more persons were hospitalized during outbreaks caused by nonpasteurized (13%) than by pasteurized dairy products (1%). This observation suggests that infections associated with nonpasteurized dairy products might be more severe, and it is consistent with the more frequent identification of bacterial, rather than viral or toxic, causative agents and with the larger proportion of illnesses affecting children.

Limitations of this analysis are primarily associated with the nature of the CDC Foodborne Disease Outbreak Surveillance System. Outbreak reporting by state and local health departments is voluntary, and outbreak reports are not always complete. For this analysis, we obtained missing data whenever possible by contacting the reporting state health department. In addition, the CDC outbreak surveillance database is dynamic; reporting agencies can submit new reports and can change or delete previous reports at any time as new information becomes available. Therefore, the results of this analysis represent data available at 1 point in time and might differ from those published earlier or subsequently.

In summary, foodborne outbreaks involving dairy products continue to be a public health problem in the United States, and this problem is disproportionately attributable to nonpasteurized dairy products. Since the US Food and Drug Administration prohibited distribution of nonpasteurized dairy products in interstate commerce for sale to consumers in 1987, all legal sale and distribution has occurred within states that permit the sale of nonpasteurized dairy products that originated in that state. How much illegal distribution in interstate commerce continues is unknown. The increased risk for outbreaks associated with legal intrastate sale of nonpasteurized dairy products demonstrated in this analysis can be weighed against the purported nutritional or other health benefits attributed to these products. Scientifically credible evidence for the health benefits of nonpasteurized dairy products beyond the benefits of those of otherwise equivalent pasteurized products is lacking (*32*). The risk for outbreaks resulting from cheese made from nonpasteurized milk in states where nonpasteurized milk sale is legal may be higher for particular groups within those states. For example, in recent years, foodborne outbreaks involving nonpasteurized dairy products have been reported in association with traditional nonpasteurized products marketed to the growing Hispanic community in the United States (*5**,**33*).

Our analysis shows that legal intrastate sale of nonpasteurized dairy products is associated with a higher risk for dairy-related outbreaks and implies that restricting sale of nonpasteurized dairy products reduces the risk for dairy-related outbreaks within that state. Pasteurization is the most reliable and feasible way to render dairy products safe for consumption. Although warning labels and signs or government-issued permits are prudent where the sale of nonpasteurized dairy products is legal, they have not been shown to be effective and, given the results of this analysis, do not seem to reduce the incidence of outbreaks involving nonpasteurized dairy products to the degree that pasteurization does (*18*). Whether certain types of warnings or more explicit health advisories might be more effective than others is unknown. Public health officials at all levels should continue to develop innovative methods to educate consumers and caregivers about the dangers associated with nonpasteurized dairy products. State officials should consider further restricting or prohibiting the sale or distribution of nonpasteurized dairy products within their states. Federal and state regulators should continue to enforce existing regulations to prevent distribution of nonpasteurized dairy products to consumers. Consumption of nonpasteurized dairy products cannot be considered safe under any circumstances.

## References

[1] US Department of Agriculture and US Department of Health and Human Services. Dietary guidelines for Americans, 2010. 7th ed. Washington: US Government Printing Office; 2010.

[2] Steele JH. History, trends, and extent of pasteurization. J Am Vet Med Assoc. 2000;217:175–8. 10.2460/javma.2000.217.17510909453

[3] US Food and Drug Administration. FDA plans to ban raw milk. In: FDA consumer. Washington: US Government Printing Office; 1987.

[4] Headrick ML, Korangy S, Bean NH, Angulo FJ, Altekruse SF, Potter ME, The epidemiology of raw milk–associated foodborne disease outbreaks reported in the United States, 1973 through 1992. Am J Public Health. 1998;88:1219–21. 10.2105/AJPH.88.8.12199702153PMC1508307

[5] Centers for Disease Control and Prevention. Outbreak of multidrug-resistant *Salmonella enterica* serotype Newport infections associated with consumption of unpasteurized Mexican-style aged cheese—Illinois, March 2006–April 2007. MMWR Morb Mortal Wkly Rep. 2008;57:432–5.18437118

[6] Centers for Disease Control and Prevention. *Salmonella* Typhimurium infection associated with raw milk and cheese consumption—Pennsylvania, 2007. MMWR Morb Mortal Wkly Rep. 2007;56:1161–4.17989645

[7] Honish L, Predy G, Hislop N, Chui L, Kowalewska-Grochowska K, Trottier L, An outbreak of *E. coli* O157:H7 hemorrhagic colitis associated with unpasteurized Gouda cheese. Can J Public Health. 2005;96:182–4.1591308010.1007/BF03403686PMC6976110

[8] Méndez Martínez C, Páez Jiménez A, Cortés-Blanco M, Salmoral Chamizo E, Mohedano Mohedano E, Plata C, Brucellosis outbreak due to unpasteurized raw goat cheese in Andalucía (Spain), January–March 2002. Euro Surveill. 2003;8:164–8.1294198210.2807/esm.08.07.00421-en

[9] Centers for Disease Control and Prevention. Multistate outbreak of *Salmonella* serotype Typhimurium infections associated with drinking unpasteurized milk—Illinois, Indiana, Ohio, and Tennessee, 2002–2003. MMWR Morb Mortal Wkly Rep. 2003;52:613–5.12844078

[10] Gillespie IA, Adak GK, O'Brien SJ, Bolton FJ. Milkborne general outbreaks of infectious intestinal disease, England and Wales, 1992–2000. Epidemiol Infect. 2003;130:461–8.12825730PMC2869982

[11] Centers for Disease Control and Prevention. Outbreak of *Campylobacter jejuni* infections associated with drinking unpasteurized milk procured through a cow-leasing program—Wisconsin, 2001. MMWR Morb Mortal Wkly Rep. 2002;51:548–9 [cited 2011 Aug 16]. http://www.cdc.gov/mmwr/preview/mmwrhtml/mm5125a2.htm.12118535

[12] McIntyre L, Fung J, Paccagnella A, Isaac-Renton J, Rockwell F, Emerson B, *Escherichia coli* O157 outbreak associated with the ingestion of unpasteurized goat's milk in British Columbia, 2001. Can Commun Dis Rep. 2002;28:6–8.11806311

[13] Health Protection Agency. Outbreaks of VTEC O157 infection linked to consumption of unpasteurized milk. Commun Dis Rep CDR Wkly. 2000;10:203, 206 10893825

[14] De Valk H, Delarocque-Astagneau E, Colomb G, Ple S, Godard E, Vaillant V, A community-wide outbreak of *Salmonella enterica* serotype Typhimurium infection associated with eating a raw milk soft cheese in France. Epidemiol Infect. 2000;124:1–7. 10.1017/S095026889900346510722123PMC2810876

[15] Villar RG, Macek MD, Simons S, Hayes PS, Goldoft MJ, Lewis JH, Investigation of multidrug-resistant *Salmonella* serotype Typhimurium DT104 infections linked to raw-milk cheese in Washington State. JAMA. 1999;281:1811–6. 10.1001/jama.281.19.181110340368

[16] Cody SH, Abbott SL, Marfin AA, Schulz B, Wagner P, Robbins K, Two outbreaks of multidrug-resistant *Salmonella* serotype Typhimurium DT104 infections linked to raw-milk cheese in northern California. JAMA. 1999;281:1805–10. 10.1001/jama.281.19.180510340367

[17] Centers for Disease Control and Prevention. Mass treatment of humans who drank unpasteurized milk from rabid cows—Massachusetts, 1996–1998. MMWR Morb Mortal Wkly Rep. 1999;48:228–9.10220255

[18] Keene WE, Hedberg K, Herriott DE, Hancock DD, McKay RW, Barrett TJ, A prolonged outbreak of *Escherichia coli* O157:H7 infections caused by commercially distributed raw milk. J Infect Dis. 1997;176:815–8. 10.1086/5173109291342

[19] Maguire H, Cowden J, Jacob M, Rowe B, Roberts D, Bruce J, An outbreak of *Salmonella* Dublin infection in England and Wales associated with a soft unpasteurized cows' milk cheese. Epidemiol Infect. 1992;109:389–96. 10.1017/S09502688000503781468523PMC2271942

[20] Maguire HC, Boyle M, Lewis MJ, Pankhurst J, Wieneke AA, Jacob M, A large outbreak of food poisoning of unknown aetiology associated with Stilton cheese. Epidemiol Infect. 1991;106:497–505. 10.1017/S09502688000675582050204PMC2271875

[21] Shiferaw B, Yang S, Cieslak P, Vugia D, Marcus R, Koehler J, Prevalence of high-risk food consumption and food-handling practices among adults: a multistate survey, 1996 to 1997. The Foodnet Working Group. J Food Prot. 2000;63:1538–43.1107969710.4315/0362-028x-63.11.1538

[22] Centers for Disease Control and Prevention. *Escherichia coli* O157:H7 infection associated with drinking raw milk—Washington and Oregon, November–December 2005. MMWR Morb Mortal Wkly Rep. 2007;56:165–7.17332727

[23] Centers for Disease Control and Prevention. *Escherichia coli* O157:H7 infections in children associated with raw milk and raw colostrum from cows—California, 2006. MMWR Morb Mortal Wkly Rep. 2008;57:625–8.18551097

[24] Lynch M, Painter J, Woodruff R, Braden C; Centers for Disease Control and Prevention. Surveillance for foodborne-disease outbreaks—United States, 1998–2002. MMWR Surveill Summ. 2006;55(SS-10):1–42.17093388

[25] Centers for Disease Control and Prevention. National Health and Nutrition Examination Survey data, 2003–2004. Hyattsville (MD): National Center for Health Statistics [updated 2008; cited 2011 Aug 16]. http://www.cdc.gov/nchs/nhanes/nhanes2003-2004/FFQRAW_C.htm#FFQ0007A

[26] Centers for Disease Control and Prevention. National Health and Nutrition Examination Survey data, 2005–2006. Hyattsville (MD): National Center for Health Statistics [updated 2008; cited 2011 Aug 16]. http://www.cdc.gov/nchs/data/nhanes/nhanes_05_06/ffqraw_d.pdf

[27] National Agricultural Statistics Service. Milk production. Washington: National Agricultural Statistics Service [updated 2011; cited 2011 Feb 22]. http://usda.mannlib.cornell.edu/MannUsda/viewDocumentInfo.do?documentID=1103

[28] Ryan CA, Nickels MK, Hargrett-Bean NT, Potter ME, Endo T, Mayer L, Massive outbreak of antimicrobial-resistant salmonellosis traced to pasteurized milk. JAMA. 1987;258:3269–74. 10.1001/jama.1987.034002200690393316720

[29] Ackers ML, Schoenfeld S, Markman J, Smith MG, Nicholson MA, DeWitt W, An outbreak of *Yersinia enterocolitica* O:8 infections associated with pasteurized milk. J Infect Dis. 2000;181:1834–7. 10.1086/31543610823796

[30] Olsen SJ, Ying M, Davis MF, Deasy M, Holland B, Iampietro L, Multidrug-resistant *Salmonella* Typhimurium infection from milk contaminated after pasteurization. Emerg Infect Dis. 2004;10:932–5.1520083510.3201/eid1005.030484PMC3323239

[31] Bradley J, Pickering LK, Jereb J. Advise families against giving children unpasteurized milk. AAP News. 2008;29:29 .10.1542/aapnews.20082912-29

[32] Potter ME, Kaufmann AF, Blake PS, Feldman RA. Unpasteurized milk: the hazards of a health fetish. JAMA. 1984;252:2048–52. 10.1001/jama.1984.033501500480206481912

[33] Centers for Disease Control and Prevention. Outbreak of listeriosis associated with homemade Mexican-style cheese—North Carolina, October 2000–January 2001. MMWR Morb Mortal Wkly Rep. 2001;50:560–2.11456331

# Research article: Nonpasteurized Dairy Products, Disease Outbreaks, and State Laws—United States, 1993–2006

**DOI:** 10.3201/eid1803.111370

**Authors:** Adam J. Langer, Tracy Ayers, Julian Grass, Michael Lynch, Frederick J. Angulo, Barbara E. Mahon

**Affiliations:** Centers for Disease Control and Prevention, Atlanta, Georgia, USA

## Medscape CME Quiz

## Earning CME Credit

To obtain credit, you should first read the journal article. After reading the article, you should be able to answer the following, related, multiple-choice questions. To complete the questions (with a minimum 70% passing score) and earn continuing medical education (CME) credit, please go to www.medscape.org/journal/eid. Credit cannot be obtained for tests completed on paper, although you may use the worksheet below to keep a record of your answers. You must be a registered user on Medscape.org. If you are not registered on Medscape.org, please click on the New Users: Free Registration link on the left hand side of the website to register. Only one answer is correct for each question. Once you successfully answer all post-test questions you will be able to view and/or print your certificate. For questions regarding the content of this activity, contact the accredited provider, CME@medscape.net. For technical assistance, contact CME@webmd.net. American Medical Association’s Physician’s Recognition Award (AMA PRA) credits are accepted in the US as evidence of participation in CME activities. For further information on this award, please refer to http://www.ama-assn.org/ama/pub/category/2922.html. The AMA has determined that physicians not licensed in the US who participate in this CME activity are eligible for *AMA PRA Category 1 Credits*™. Through agreements that the AMA has made with agencies in some countries, AMA PRA credit may be acceptable as evidence of participation in CME activities. If you are not licensed in the US, please complete the questions online, print the certificate and present it to your national medical association for review.

## Article Title: Nonpasteurized Dairy Products, Disease Outbreaks, and State Laws—United States, 1993–2006

## CME Questions

**1. Your patient is a 12-year-old girl who presents for 3 days of gastroenteritis symptoms. Her father reports that most of the family has been consuming unpasteurized dairy products for the past year, and he is concerned that his children are at risk for foodborne illness.On the basis of the current study by Langer and colleagues, what should you consider regarding the epidemiology of foodborne illness related to dairy products?**A. Fewer than 20 cases were reported to the CDC between 1993 and 2006B. Unpasteurized dairy products accounted for most outbreaksC. Yogurt was the dairy product implicated in most casesD. Unpasteurized cheese was more likely to promote infection compared with unpasteurized milk**2. In your management of this patient, what should you consider from data reported in the current study regarding the clinical picture of infection associated with contaminated dairy products?**A. The rate of hospitalization was similar whether the infection was due to pasteurized or unpasteurized productsB. The overall hospitalization rate was 25%C. The case-mortality rate approached 50% for infections due to unpasteurized productsD. Patients with infection due to unpasteurized products were younger compared with those with infection due to pasteurized products**3. What was the most common organism isolated from patients with infection due to consumption of unpasteurized dairy products in the current study?**A. NorovirusB. *Salmonella* spp.C. *Escherichia coli*D. *Campylobacter* spp.**4. The patient's mother drinks pasteurized milk, and she, too, is not feeling well. What can you tell her was the most common source of contamination of pasteurized dairy products in the current study?**A. Infected food handlersB. Temperature abuseC. Consumption of dairy products beyond their expiration dateD. Poor pasteurization practices

## Activity Evaluation

**Table:** 

1. The activity supported the learning objectives.				
Strongly Disagree				Strongly Agree
1	2	3	4	5
2. The material was organized clearly for learning to occur.				
Strongly Disagree				Strongly Agree
1	2	3	4	5
3. The content learned from this activity will impact my practice.				
Strongly Disagree				Strongly Agree
1	2	3	4	5
4. The activity was presented objectively and free of commercial bias.				
Strongly Disagree				Strongly Agree
1	2	3	4	5
